# Memory Consolidation in the Cerebellar Cortex

**DOI:** 10.1371/journal.pone.0011737

**Published:** 2010-07-29

**Authors:** Daniel O. Kellett, Izumi Fukunaga, Eva Chen-Kubota, Paul Dean, Christopher H. Yeo

**Affiliations:** 1 Department of Neuroscience, Physiology and Pharmacology, University College London, London, United Kingdom; 2 Department of Psychology, University of Sheffield, Sheffield, United Kingdom; University of Queensland, Australia

## Abstract

Several forms of learning, including classical conditioning of the eyeblink, depend upon the cerebellum. In examining mechanisms of eyeblink conditioning in rabbits, reversible inactivations of the control circuitry have begun to dissociate aspects of cerebellar cortical and nuclear function in memory consolidation. It was previously shown that post-training cerebellar cortical, but not nuclear, inactivations with the GABA_A_ agonist muscimol prevented consolidation but these findings left open the question as to how final memory storage was partitioned across cortical and nuclear levels. Memory consolidation might be essentially cortical and directly disturbed by actions of the muscimol, or it might be nuclear, and sensitive to the raised excitability of the nuclear neurons following the loss of cortical inhibition. To resolve this question, we simultaneously inactivated cerebellar cortical lobule HVI and the anterior interpositus nucleus of rabbits during the post-training period, so protecting the nuclei from disinhibitory effects of cortical inactivation. Consolidation was impaired by these simultaneous inactivations. Because direct application of muscimol to the nuclei alone has no impact upon consolidation, we can conclude that post-training, consolidation processes and memory storage for eyeblink conditioning have critical cerebellar cortical components. The findings are consistent with a recent model that suggests the distribution of learning-related plasticity across cortical and nuclear levels is task-dependent. There can be transfer to nuclear or brainstem levels for control of high-frequency responses but learning with lower frequency response components, such as in eyeblink conditioning, remains mainly dependent upon cortical memory storage.

## Introduction

Several forms of learning are clearly dependent upon the cerebellum but there is uncertainty as to how the neural plasticity that underpins them is distributed within and outside the cerebellar circuitry. Modification of gain in the vestibulo-ocular reflex (VOR) involves plasticity both in the cerebellar cortex and in Purkinje cell target neurons in the brainstem vestibular complex [1]–[5] and may be representative of other forms of cerebellum-dependent motor learning [6]. But a recent model suggests that the distribution of plasticity across the cortical and brainstem (or cerebellar nuclear) levels depends upon the nature of the task and is related to the magnitude of the central delays in the error signal, the mechanical properties of the motor plant (the muscles, the hard and soft tissues connected to them and the effector structure) and to the component frequencies of the learned responses [7]. For VOR modification, transfer of learning from cortex to brainstem is required only for responses in the higher frequency range. Delay classical conditioning of the eyeblink and nictitating membrane response (NMR) of rabbits is a form of cerebellum-dependent learning with task requirements very different from those of VOR modification. Its conditioned responses (CRs) are relatively slow, suggesting that memory storage for NMR conditioning may be predominantly cortical. Here we have used reversible inactivation of critical parts of the cerebellar circuitry to analyze the distribution of plasticity across cerebellar cortical and nuclear levels.

Specific territories in the inferior olive, the cerebellar cortex and the cerebellar nuclei are essential for normal NMR conditioning. They form an olivo-cortico-nuclear compartment that includes the face somatosensory regions of the dorsal accessory olive, eyeblink microzones in the cortical C1, C3 or D0 zone, especially those within cortical lobule HVI, and related projection areas in the anterior interpositus nucleus [8]. If given before conditioning begins, discrete, reversible inactivations or disturbances of normal transmission at any level in the compartment effectively prevent acquisition of new conditioned responses (CRs) [9]–[11]. When made after conditioning has been established, similar interventions impair or prevent the expression of CRs [10], [12], [13], and see [14], [15] for reviews. As we have suggested, the uniformity of these inactivation effects relates to the presence of an inhibitory, nucleo-olivary feedback loop [16]–[17] that helps maintain dynamic states at each level in the compartment. Thus, interventions at any level in the compartment have consequences for activity at the other levels and produce comparable disturbances of acquisition and expression that do not directly localize memory storage to any particular level.

**Figure 1:**
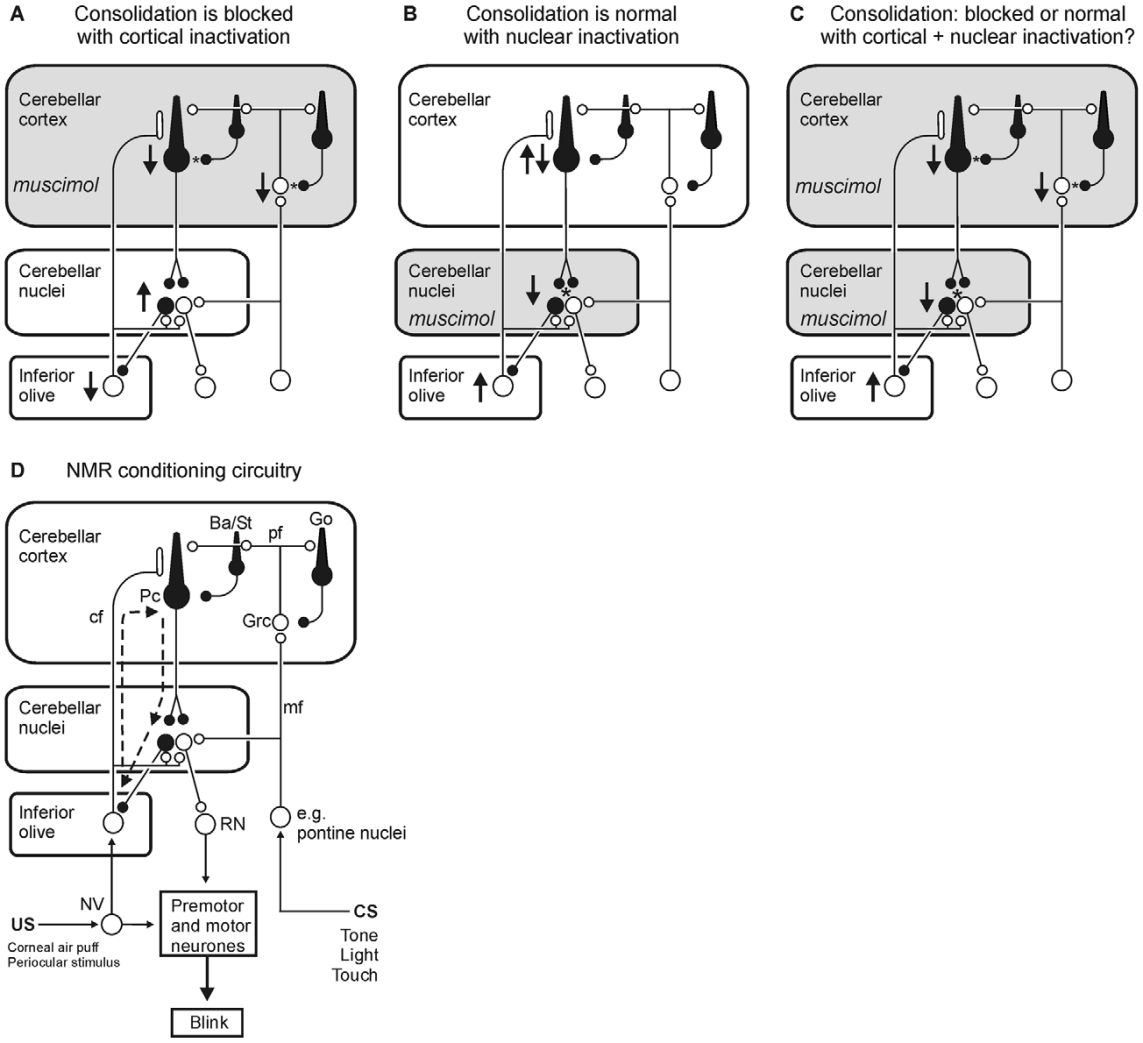
Effects of local inactivations on olivocorticonuclear neuronal activity and memory consolidation. Simplified views of olivocorticonuclear circuitry involved in motor memory formation, with cortical interneurons, multiple mossy fiber inputs, and some brainstem circuits omitted for clarity. Each panel shows how information transmission and excitabilities within the system may change after different interventions. Excitability increases (↑) and decreases (↓) are indicated. Asterisks denote synapses at which muscimol may be acting. Post-training muscimol infusions to cerebellar cortex (A) prevent consolidation, but it is uncertain whether consolidation processes are disrupted directly in the targeted structure or indirectly through disturbance of the OCN loop. In particular, the excitability of cerebellar nuclear neurons will increase as a consequence of cortical inactivation. However, post-training muscimol infusions to the cerebellar nuclei (B) do not affect consolidation. Nucleo-olivary inhibition is depressed, so olivary excitability will be increased. At Purkinje cells, increased climbing fiber activity increases complex spike activity with a corollary reduction in simple spike activity, indicated by ↑↓, but this does not impair consolidation processes. It remains possible that consolidation occurs entirely in the cerebellar nuclei only if these processes are disrupted by excitability increases, but insensitive deep neuronal inhibition with muscimol. Hence in the present investigation (C), post-training muscimol infusions to both cortex and nuclei cause a deep inhibition of cortical neurons, whilst protecting the cerebellar nuclei from disinhibition. (D) A key to panels A–C and a model of cerebellar pathways engaged in NMR conditioning. CS- and US-related information converges within the cerebellar cortex and within the cerebellar nuclei through mossy fiber and climbing fiber inputs, respectively. The inhibitory olivo-cortico-nuclear loop (OCN) is indicated by dashed arrows. *Conventions*: excitatory neurons and synapses are shown in white; inhibitory neurons and synapses in black. Abbreviations: Ba, basket cell; cf, climbing fiber; Go, Golgi cell; Grc, granule cell; mf, mossy fibers; NV, trigeminal nucleus; Pc, Purkinje cell; pf, parallel fibers; RN, red nucleus; St, Stellate cell.

Cerebellar cortical and nuclear mechanisms in learning were recently dissociated by analyzing memory consolidation. Reversible, post-training inactivations of lobule HVI, using the GABA_A_ agonist muscimol, significantly disrupted consolidation of NMR conditioning whereas similar inactivations of critical regions of the anterior interpositus nucleus (AIP) did not [18], [19] suggesting that these memories are consolidated and stored within the cerebellar cortex. However, there is another possibility (see Figure 1). Cerebellar cortical muscimol depresses Purkinje cell activity with a consequent disinhibition of nuclear neurons whereas direct application of muscimol to cerebellar nuclear neurons hyperpolarizes them and their activity drops sharply [20]. Thus, memory formation for eyeblink conditioning might not be cortical, but nuclear and sensitive to increases, but not decreases, in firing rate [19].

Here we resolve the cerebellar consolidation question using simultaneous, post-training inactivations of both the cerebellar cortex *and* nuclei. If consolidation is predominantly nuclear and sensitive to raised nuclear excitability caused by cortical inactivation, then it would be protected by the additional nuclear inactivation. If it is intracortical, then the cortical inactivation would still impair consolidation even with simultaneous nuclear inactivation. We found that simultaneous inactivation of the cerebellar cortex and nuclei immediately after each of four training sessions strongly impaired consolidation, suggesting a major intracortical consolidation process in this simple associative learning.

## Results

### Experimental Groups

**Figure 2:**
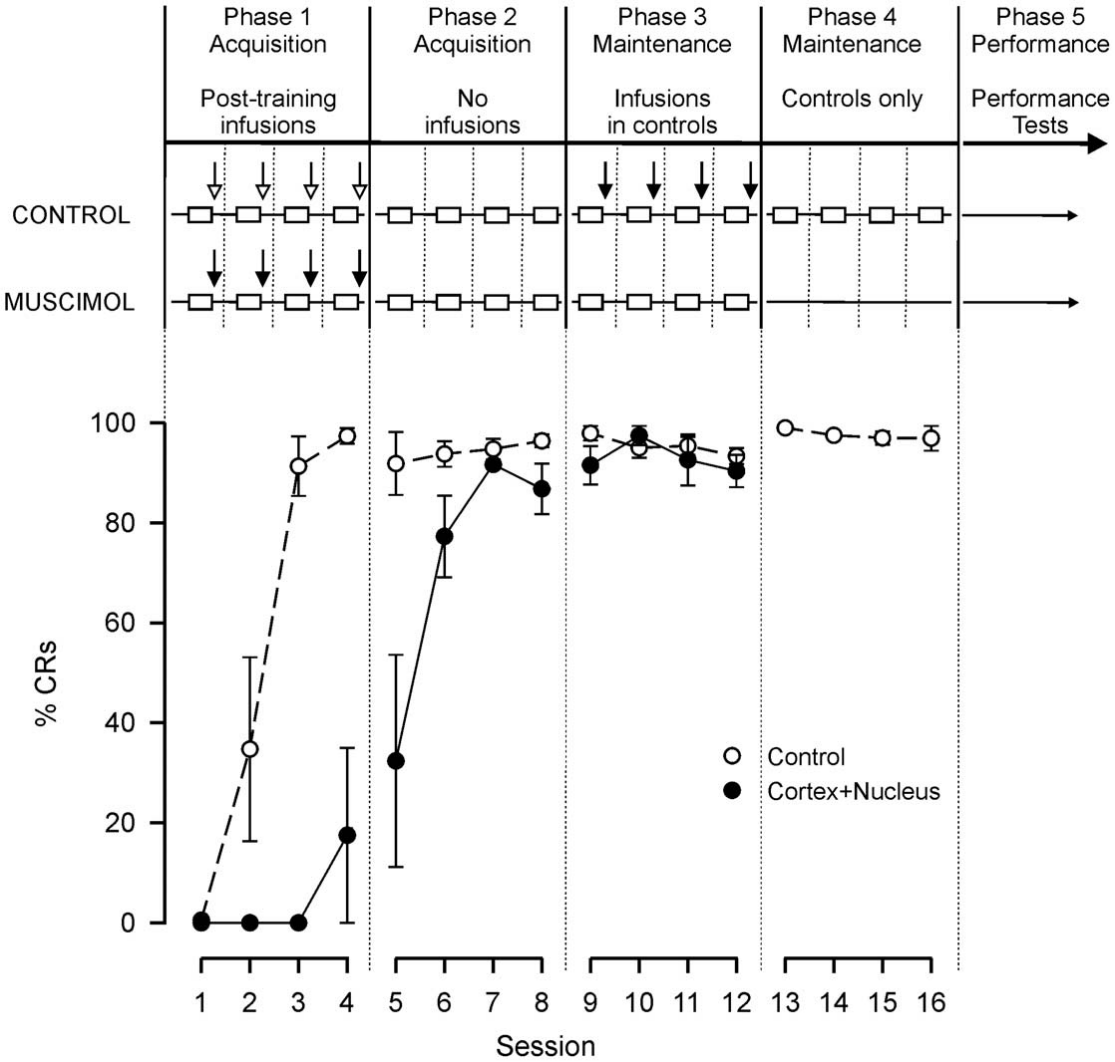
Experimental design and effects of post-training muscimol infusions to cerebellar cortex and nuclei on consolidation. *Experimental design*: each daily session is shown as an open rectangle. Solid vertical lines indicate 3 day rest periods. Post-training cortical and nuclear infusions of muscimol (closed arrows) or vehicle (open arrows) are indicated. *Behavioral data*: daily, mean session %CRs (±1 SEM) for the Control (cortical and nuclear vehicle, n = 4) and Cortex+Nucleus (cortical and nuclear muscimol, n = 4) groups. Control subjects acquired asymptotic CRs during Phase 1, but Cortex+Nucleus subjects did not. Cortex+Nucleus subjects developed robust CRs during Phase 2, when muscimol was not given. Post-training infusions of muscimol given to the Control subjects during Phase 3 had no consequences for the maintained expression of CRs during Phases 3 and 4.

39 rabbits were each implanted with two guide cannulae: one guide was directed to eyeblink control regions in cerebellar cortical lobule HVI and the other was directed to the anterior interpositus nucleus (AIP). 31 of these subjects received post-training muscimol infusions in cortex and nuclei. 8 subjects formed a control set and received post-training cortical and nuclear infusions of saline vehicle. All post-training infusions were made within 5 minutes of the end of each session. Each Phase of training consisted of a four, daily training sessions of 50 trials each and there was an interval of three days with no training between each Phase of training (see Figure 2 for an illustration of the experimental design). These protocols are similar to those used in our earlier study of consolidation following individual inactivations of the cerebellar cortex or nuclei [18]. After final performance testing and histological verification (see Methods), each of the muscimol infused subjects was allocated to one of the following groups (see Performance Testing and Histology results below for full details of group allocations): Cortex+Nucleus (muscimol inactivations at the cortical and nuclear sites both fully effective, *n* = 4), Incomplete (muscimol inactivations at the cortical site partially effective and at the nuclear site fully effective, *n* = 6), Nucleus Only (muscimol inactivation effective only at the nuclear site, *n* = 7), Off-Target (muscimol inactivation incomplete at cortical and nuclear sites, *n* = 10). 4 of these 31 subjects were excluded from further analysis due to guide cannula-related damage revealed by histology. Of the 8 saline vehicle-infused subjects, 4 were admitted to the Control group, and the remaining 4 were excluded due to off-target cannula placement as revealed by Performance Testing.

### Post-training simultaneous inactivation of cortex and nuclei impairs consolidation

**Figure 3:**
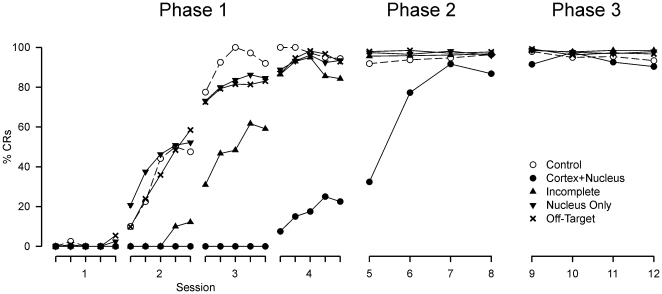
Incomplete and off-target infusions confirm a critical role of cerebellar cortex in consolidation. Expanded view of CR acquisition in Phase 1 (mean CR frequencies for successive 10-trial blocks of Phase 1 in all experimental groups, followed by mean session frequencies for each session of Phase 2 and 3). Rate of acquisition is related to depth of cortical inactivation with muscimol: Cortex+Nucleus (n = 4) subjects fail to acquire robust CRs during Phase 1, whilst Incomplete (n = 6) subjects do acquire CRs, but not as rapidly as Nucleus Only (n = 7) and Off-Target (n = 10) subjects, which acquire CRs at a similar rate to Controls (n = 4).

All subjects Subjects in the Cortex+Nucleus group with successful, simultaneous inactivation of the cortex and nuclei immediately after each training session of Phase 1 (sessions 1–4) failed to acquire robust CRs during this Phase (see Figures 2 and 3): 3 of 4 subjects expressed no CRs during these first 4 sessions. In contrast, all subjects in the Control group began to express CRs on Session 2, and were asymptotic (>90% CRs) on Session 4. Similarly, subjects in the Incomplete group all reached asymptote by Session 4 (Figure 3) but, during Session 2, only 2 of 6 subjects expressed any CRs. All subjects in the Nucleus Only and Off-Target groups were asymptotic at Session 4 (Figure 3): During Session 2, CRs were already developing in 5 out of 5 of the Nucleus Only, and 9 out of 10 of the Off-Target subjects.

#### Group Analysis 1 (all groups, Phase 1)

Comparison of all groups during Phase 1 (see Figure 3) revealed a significant difference between groups (Kruskal-Wallis 1-way ANOVA, H = 30.01, df = 4, *P*<0.001). The Cortex+Nucleus group expressed significantly fewer CRs during Phase 1 compared to the Control, Nucleus Only, and Off-Target groups (Dunn's *post hoc* test, all comparisons *P*<0.05). The Incomplete group learning rate is intermediate between those of the Cortex+Nucleus and other groups and does not differ significantly from them (Dunn's *post hoc* test, all comparisons *P*>0.05).

#### Group Analysis 2 (all groups, Phase 2)

Training was continued in the absence of muscimol infusions during Phase 2 (see Figure 3). The Cortex+Nucleus group expressed fewer CRs than the Incomplete, Nucleus Only and Off-Target groups (Kruskal-Wallis 1-way ANOVA, H = 33.13, df = 4, *P*<0.001, Dunn's test *P*<0.05) but approached asymptote on Session 7. All other group differences were non-significant (Dunn's test *P*>0.05).

#### Group Analysis 3 (Muscimol group, Phase 2 vs. all other groups, Phase 1)

After Phase 1, where the Cortex+Nucleus group had acquired few CRs, learning in Phase 2 was at a rate comparable to that of the Control group in Phase 1 (see Figure 3). The frequency of CRs expressed by the Cortex+Nucleus group in Phase 2 was not significantly different from that of all other groups in Phase 1 (Kruskal-Wallis 1-way ANOVA, H = 7.56, df = 4, *P* = 0.11). Nonetheless, inspection of the learning curves shown in Fig. 2 suggests a clear trend towards some savings in the Cortex+Nucleus group. The magnitude of the savings is approximately equivalent to the value of one training session (see Discussion for further details).

#### Group Analysis 4 (Control group, Sessions 5–16)

To reveal whether there was potentially some normal learning in the Cortex+Nucleus group during Phase 1 but that such learning is masked or otherwise disturbed by general and cumulative effects of muscimol treatments that might extend into the succeeding training sessions, muscimol was re-infused in Control group subjects in Phase 3 (see Figures 2 and 3). Analysis of sessions 5–16 (Phases 2–4) within Control subjects revealed no significant change in CR frequency (Friedman 1-way repeated measures ANOVA, χ^2^ = 11.73, df = 11, *P* = 0.39), indicating that muscimol had no effect on the maintenance of established CRs during Phase 3, and no subsequent, longer term effects during Phase 4.

### Performance Testing and Localization of Infusions

Reinfusions of muscimol and, in separate sessions, the competitive AMPA/kainate receptor antagonist 6-cyano-7-nitroquinoxaline-2,3-dione (CNQX) at the cortical and nuclear sites in the final, Phase 5 of the experiment were used to assess the proximity of the infusion sites to the critical control regions. Successful block of both sites in Phase 5 was taken as evidence that the blocks in Phase 1 had been similarly successful. First, rapid and deep depressions of CR frequency following individual infusions of muscimol in the cortex and in the nuclei, was necessary evidence for effective blocks at both sites. Second, rapid and deep depression of CR frequency to CNQX infusion in the cortical, but not in the nuclear, locations was additionally required to confirm the completeness of the cortical muscimol infusions. For an explanation of the logic of this experimental design, see the Methods.

#### (i) Infusion of 7 nmol muscimol to cortex – see Figure 4A

In the 4 subjects admitted to the Cortex+Nucleus group, cortical muscimol produced a rapid and prolonged loss of CR expression, with CRs reduced to 8.3±2.8% (mean ± SEM) at 50 min. All 4 of these subjects expressed 0% CRs at 2 and 4 hrs after muscimol, but with full recovery at 24 hrs. In the 4 subjects admitted to the Control group, cortical muscimol reduced CRs to 42.5±24.6% after 50 min, and to 10.8±10.8% at 2 hrs. In those subjects admitted to the Incomplete, Nucleus and Off-Target groups, CR frequency at 50 min was 28.3±18.3%, 47.9±17.0%, and 37±14.9%, respectively. There was no significant difference between CR frequencies at 50 min across groups (Kruskal-Wallis 1-way ANOVA, H = 0.62, df = 4, *P* = 0.96).

**Figure 4:**
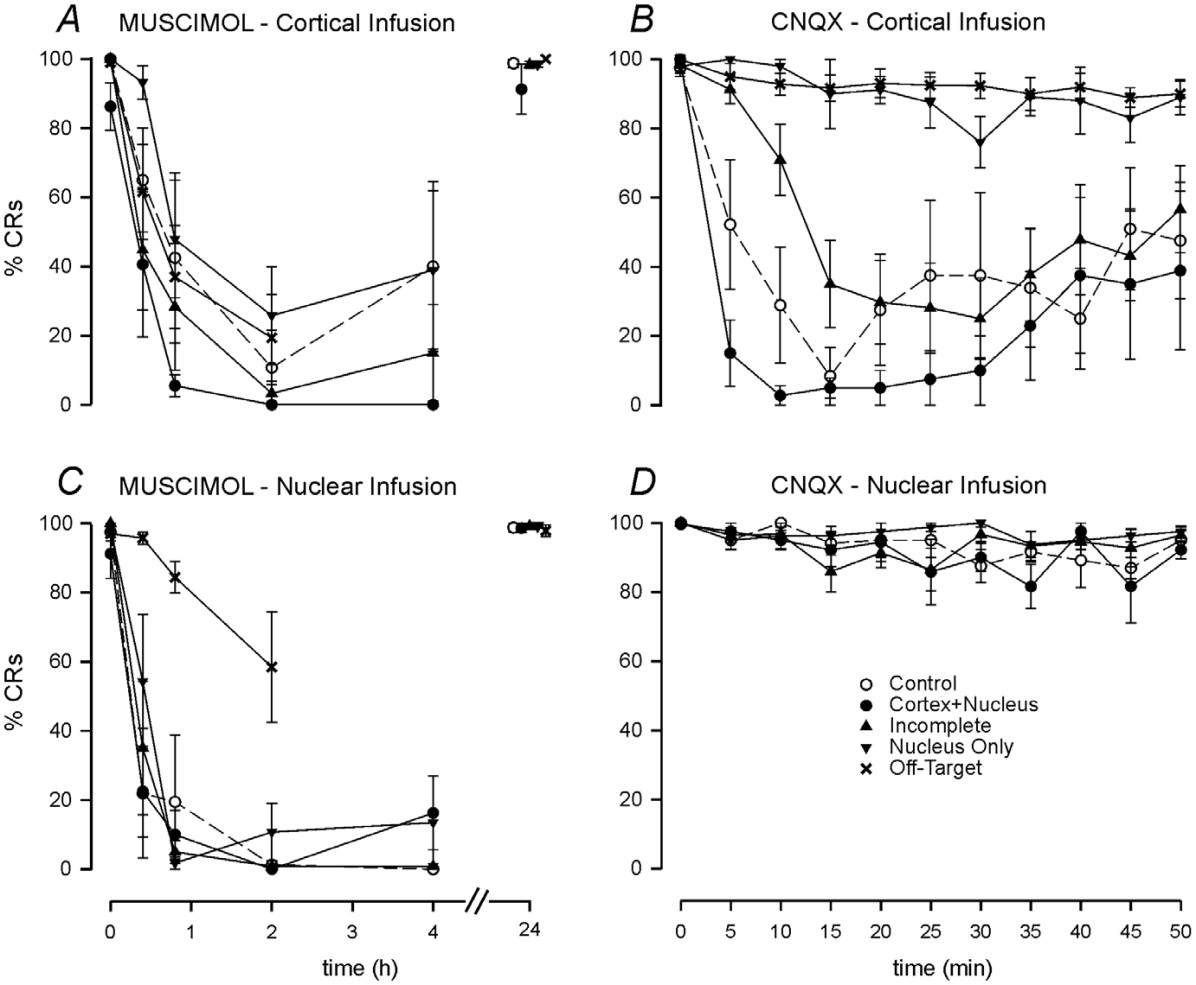
Effects of cortical and nuclear muscimol and CNQX on performance of established CRs. Mean (±1 SEM) effects of muscimol (7 nmol; left panels) and CNQX (6 nmol; right panels) on CR performance when infused into cortical (2 µl; top panels) or nuclear cannulae (1 µl; bottom panels). Only the Cortex+Nucleus subjects satisfy all the criteria for inclusion in this principal group.

#### (ii) Infusion of 7 nmol muscimol to nuclei – see Figure 4C

In the Cortex+Nucleus group, muscimol to the nuclei produced a rapid and prolonged loss of CR expression. In all these subjects, the first 10-trial block with 0% CRs occurred more quickly than after cortical muscimol. Thus, it was established that at no time would the cortex have been inactivated before the nuclei in Phase 1 of the experiment and so there would have been no short period of nuclear disinhibition to confound the findings. At 50 min the mean CR frequency was 10.0±7.1% (mean ± SEM), and at 2 hrs all subjects were at 0%. At the 50 min and 2 hr time points, the Control group similarly had mean CR frequencies of 19.4±19.4%, and 1.3±1.3%, respectively. The Incomplete and Nucleus groups had comparable CR frequencies at 50 min of 5.0±3.4% and 1.8±1.8%, respectively, whilst the Off-Target group remained at 84.5±4.6%. Group CR frequencies at the 50 min time point were significantly different (Kruskal-Wallis 1-way ANOVA, H = 21.54, df = 4, *P*<0.001), with the Off-Target group expressing significantly more CRs than the Control, Incomplete, and Nucleus groups (Dunn's test, *P*<0.05).

#### (iii) Infusion of 6 nmol CNQX to cortex – see Figure 4B

Infusion of CNQX via the cortical cannulae tested whether muscimol infusions via this route had exerted their effects upon the eyeblink control region of HVI, as intended, rather than by migration to the cerebellar nuclei. In the Cortex+Nucleus group, cortical CNQX abolished CRs within the first or the second 10-trial block (see Figure 4). In the Control group, 2 of 4 subjects reached 0% CRs in the second block of training, with a group mean of 28.9±16.7% (mean ± SEM) at 10 min. Of the Incomplete group, only 3 of 6 subjects achieved a 0% block at various time points ≥15 min. The Control group mean CR frequency in the second block was 70.9±10.3%, and for the Nucleus and Off-Target groups, it was 98.0±2.0% and 92.8±3.2%, respectively. Group CR frequencies in the second block were significantly different (Kruskal-Wallis 1-way ANOVA, H = 18.38, df = 4, *P* = 0.001). Compared to the Nucleus group, both the Cortex+Nucleus and the Control groups expressed significantly fewer CRs, with the Cortex+Nucleus group also at significantly lower levels than the Off-Target group (Dunn's test, *P*<0.05). Control and Cortex+Nucleus groups were not significantly different.

#### (iv) Infusion of 12 nmol CNQX to cerebellar nuclei – see Figure 4D

Infusion of CNQX to nuclei had no significant effect on CR expression, consistent with our earlier observations [18]. In the second block there were no significant differences between groups (Kruskal-Wallis one-way ANOVA on ranks, H = 2.99, df = 3, *P* = 0.39).

#### (v) Performance Testing Summary

Taken together, cortical and nuclear CNQX testing differentiates those subjects that received muscimol confined to cerebellar cortex from those in which cortical muscimol infusions clearly diffused to the cerebellar nuclei. Critically, these latter subjects included those with a block of CR expression during Performance Testing but with cerebellar cortical inactivations insufficient to prevent consolidation during Phase 1. Consistent with this classification, several subjects in the Nucleus and Off-Target groups showed delayed CR impairment after cortical muscimol during Performance Testing and histological reconstruction of the infusion sites suggests that these infusions, intended to affect cortical eyeblink regions, exerted their effects by migration to the cerebellar nuclei.

Although there were no significant differences between the Cortex+Nucleus and Control groups during muscimol and CNQX testing, there is a trend towards the muscimol group having more effective cortical placements. Thus, in principle, the consolidation impairments of the Cortex+Nucleus group might really depend upon general factors discussed in the experimental design logic (see Methods (v) - Performance Testing). This possibility can be rejected. First, the Incomplete group had substantially weaker performance blocks than the Control group (see Figure 4) and yet they showed a clear trend towards consolidation impairment. Second, in our previous study, where single, cortical cannulations allowed exact matching of performance deficits in the Cortex+Nucleus and Control groups, Control group subjects showed no muscimol-related general decrements in CR expression through Phases 4 and 5.

#### (vi) Histology

**Figure 5:**
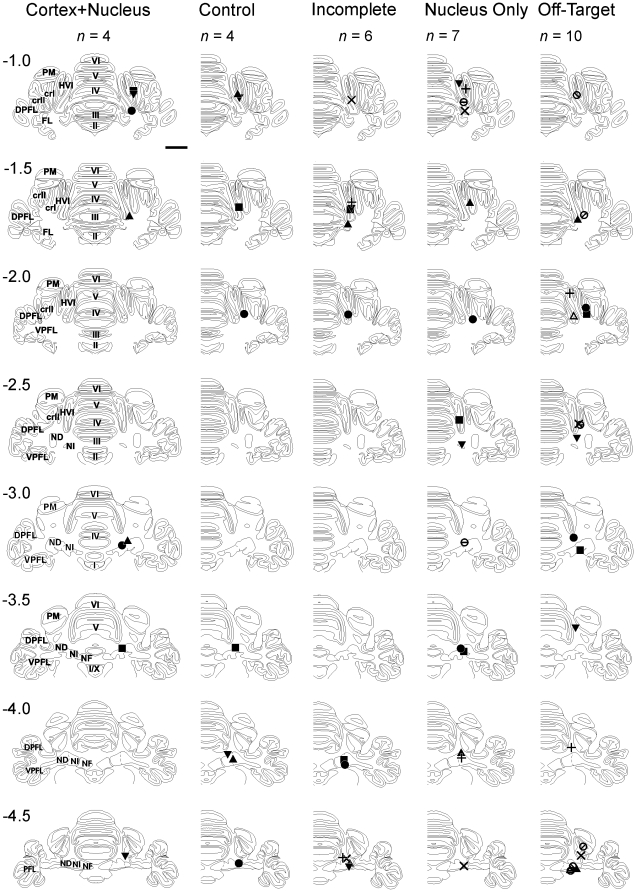
Histological reconstruction of cortical and nuclear infusion sites. Cannula tip locations are shown for all subjects in each group on a series of 8 standard transverse sections at levels from −1.0 mm to −4.5 mm relative to skull lambda. For each subject in the five groups, two matching symbols indicates the location of the cortical and nuclear cannula tips. For example, one subject in the Cortex+Nucleus group has cannula positions indicated by filled circles – the cortical placement is seen at level −1.0 mm and the nuclear placement at level −3.0 mm. The scale bar indicates 5 mm. The separation of the two cannula tip locations may be judged by reference to distances in the rostro-caudal axis and transverse planes. *Abbreviations*: crI and crII - crus 1 and 2 (of ansiform lobe); DPFL - dorsal paraflocculus; FL - flocculus; HIV-V, HVI - hemispheral lobules 4–5 and 6 (of Larsell); ND - dentate nucleus; NF - fastigial nucleus; NI - interpositus nucleus; PM - paramedian lobe; VPFL - ventral paraflocculus; II-X vermis lobules 2–10 (of Larsell).

Nissl-stained cerebellar sections were examined under a light microscope to identify the location of the injector tip and whether there was incidental damage. In the Cortex+Nucleus group, the cortical cannulae were located within lobule HVI, either in the lateral lobule (*n* = 2) or in the ventral portion of the medial lobule (*n* = 2) and their nuclear cannulae were entirely within, or in the vicinity of, the interpositus nucleus. Reconstructions of all cannula positions are shown in Figure 5. Control group subject cannulae were in locations comparable with those of the Cortex+Nucleus group. In the Incomplete group, cortical cannulae placements were less satisfactory: they lay either close to a fissure or within lobule V but the nuclear cannulae, were well placed. In the Nucleus Only and Off-Target groups, cortical cannulae were mostly within a fissure, or within white matter, raising the likelihood of spread to the nuclei by either route without significant cortical retention of the drug. Nuclear cannulae in the Nucleus group were satisfactory, but were mostly misplaced in the Off-Target group, with several close to or in the IV^th^ ventricle.

**Figure 6:**
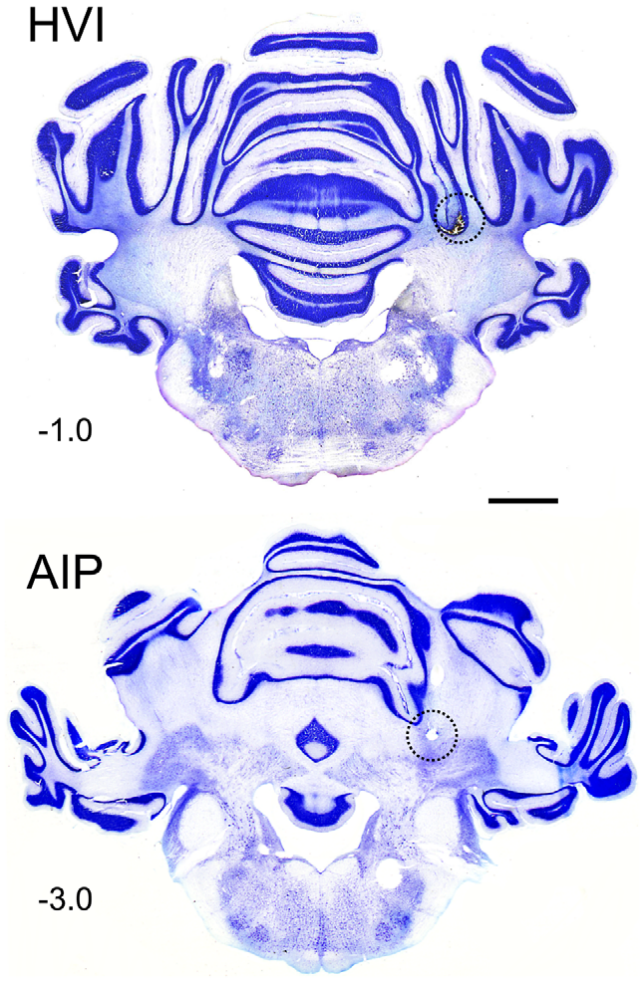
Histological identification of cannula tip and spread of infusion. Photomicrographs of 50 µm transverse cerebellar sections from a experimental subject from the Cortex+Nucleus group. Sections are stained with cresyl violet, showing location of cortical (HVI) and nuclear (AIP) cannula tips (arrowhead). Positions relative to skull λ are indicated (mm). The approximate extent of diffusion of pontamine sky blue dye (2 µl for cortex, 1 µl for nuclei) was plotted on adjacent neutral red-stained sections, and superimposed on the current sections (dotted lines). Calibration bar represents 2 mm.

4 subjects that had received muscimol infusions, and 4 subjects that had received saline infusions in Phase 1, were excluded due to cannula-related damage (substantial gliosis, and/or Purkinje or granule cell loss). None of the subjects in the Muscimol group had significant incidental damage. Photomicrographs of the cannulae placements in a subject from this group are shown in Figure 6.

#### (vii) Conditioned Response topography

**Figure 7:**
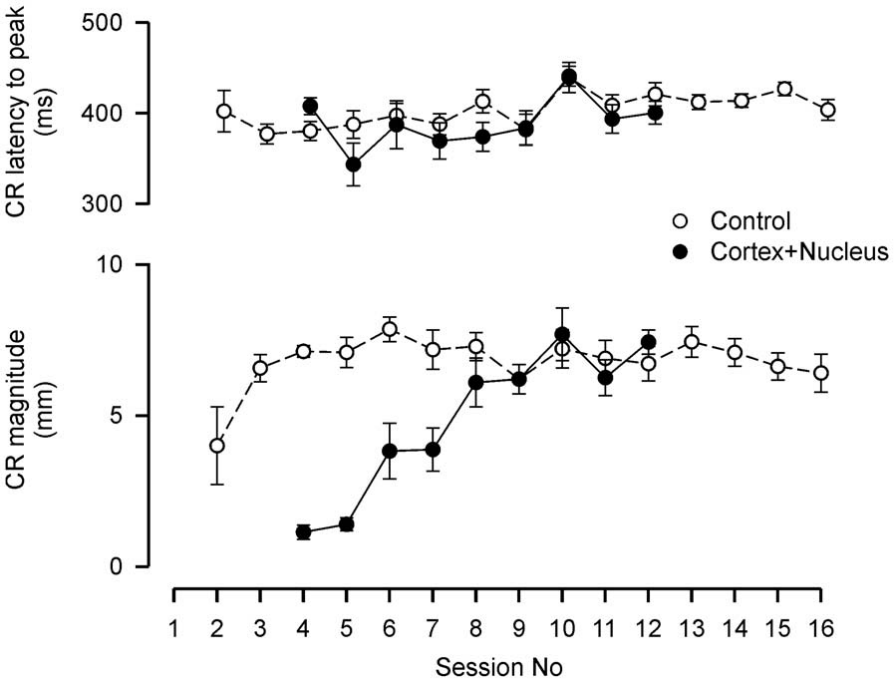
Conditioned response profiles. Mean (±1 SEM) CR latency to peak, and magnitude (amplitude above threshold of 0.5 mm), in Control and Cortex+Nucleus subjects.

As a further functional indicator of potential cerebellar damage in the Cortex+Nucleus and Control groups, CR latency-to-peak and magnitude were examined throughout Phases 1–4 (see Figure 7). There was no significant change in CR latency-to-peak across sessions in the Control group (one-way ANOVA; *F* = 1.36, df = 14, *P* = 0.22) or Cortex+Nucleus group (*F* = 1.22, df = 8, *P* = 0.34). Likewise, Control and Cortex+Nucleus group CRs developed in magnitude at a comparable rate: on session 8, Control CR magnitude was 7.3±0.5 mm (mean ± SEM). This session is equivalent to session 12 in Cortex+Nucleus subjects (*i.e.* the 8^th^ drug-free session), in which the CR magnitude was 7.4±0.4 mm. Thus, in both groups, CRs were of similar magnitude and well-timed throughout, with their peaks coinciding with the US, suggesting that there was no major incidental damage in these subjects.

### Frequency content of CR drive

We have suggested that the relative dependence of cerebellum-dependent forms of learning on cortical and nuclear mechanism may relate to the frequency component content of the learned responses. Responses with lower frequency components would always be more dependent upon cortical mechanisms. So we analyzed the frequency composition of the CR drive. For the ISI of 350 ms used here, CR profiles are single-peaked with a typical duration of at least 400 ms. Our previous analysis of NM position and retractor bulbi EMG suggests that this response derives from a neural control signal to the motoneuron pool that has a Gaussian profile, and for an ISI of 350 ms the standard deviation of the Gaussian is about 130 ms (see [21], Figure 11). Fourier analysis (details in Methods) of such a Gaussian reveals the frequency components of the drive signal, to show that 95% of its frequency content is below 3.7 Hz. This frequency limit is low compared to that for the primate VOR (20–25 Hz, see [7]).

## Discussion

Post-training, simultaneous, reversible inactivations of the cerebellar cortex and cerebellar nuclei have here produced impairments in the consolidation of classical NMR conditioning comparable with those produced previously by cortical inactivations alone [18]. These consolidation deficits cannot have been due to disinhibition of the cerebellar nuclei following cortical muscimol application since the deep nuclei themselves were deeply inhibited and thus protected from disinhibition by simultaneous application of muscimol directly to them. Because such post-training nuclear inactivations alone have no effects upon consolidation [18], the present results indicate that an essential component of NMR conditioning consolidation is intracortical. Furthermore, the inactivations would have prevented cortico-nuclear information exchange during the inactivation period of at least 4 hours, so a system consolidation mechanism, whereby a temporary cortical trace might be transmitted to the cerebellar nuclei, is ruled out within this particular post-training time window. But a putative system consolidation mechanism involving transfer within the layers of the cerebellar cortex, for example from granule cells to Purkinje cells, or from cortical interneurons to Purkinje cells, certainly could have been disrupted by the inactivation.

Although there is a wealth of experimental evidence and theoretical work pointing to cerebellar cortical mechanisms in memory formation (see [1], [22], [23], for reviews), we suggest that the current analysis of memory consolidation is the first to confirm that a critical part of memory storage for eyeblink/NMR is normally cortical. But in reaching this conclusion it is important to recognize that the identification of intracortical memory storage does not rule out additional memory storage at other levels in the olivo-cortico-nuclear compartment. Our interventions have been successful in disrupting a cortical component of memory storage – other pharmacological tools or alternative interventions during the post-training period might reveal subsidiary storage at other locations.

It should be noted that although the consolidation deficits produced by cortical inactivation alone [18] and those produced here by simultaneous cortical and nuclear inactivation are significant and very substantial, they are not complete. If consolidation had been completely prevented in either experiment, then sessions 1–4 of Phase 1 would have produced no detectable learning and the learning curves for the Cortex+Nucleus group would be offset from those for the Controls by a full 4 sessions. For cortical inactivation alone, the offset is closer to 3 sessions (see [18], Figure 2) as judged by the number of sessions to about 40% CRs and to asymptote. For the cortical and nuclear inactivations here, Figure 2 reveals that the offset is also approximately 3 sessions judged by the number of sessions to the same two performance points (overall learning rates are somewhat faster in the current experiment which uses the notably placid Murex strain of rabbit). Thus, post-training inactivation of the ipsilateral cerebellar cortex is seen to produce major and significant impairments of conditioning but some consolidation can still occur, consistent with an earlier observation that fully effective, pretraining, ipsilateral cortical inactivations produce major, but subtotal, loss of acquisition [24]. There are several possible interpretations. First, the inactivations may be incomplete, so consolidation might indeed be entirely within the ipsilateral cerebellar cortex. Second, a component of memory storage might depend upon the contralateral cerebellar cortex, since bilateral cortical lesions can produce more profound losses of NMR acquisition/performance [25]. Third, some form of stabilization of the memory trace may take place within the training session itself and before the inactivations are applied. Fourth, there may be extracerebellar storage in, for example, motor cortical areas. This possibility may be less relevant for rabbit NMR conditioning, which is our model here and which is extremely cerebellum-dependent. However, such extracerebellar mechanisms may come into play for conditioning of the external eyelid blink, which has considerable baseline and voluntary movements especially in other species (see [22], [26], [27] for reviews). Fifth, there may be subsidiary plasticity in the cerebellar nuclei, a proposition that has attracted considerable theoretical and experimental interest.

In considering additional, nuclear memory storage in eyeblink/NMR conditioning there have been two main lines of enquiry. First, there is ultrastructural evidence for conditioning-related increased excitatory synaptic number in the cerebellar nuclei in rats [28] and second, a series of experiments using cortical lesions or the application of GABA_A_ antagonists to the cerebellar nuclei [29]–[31] reveal a short-latency, conditioning-specific response to the CS when cortical inhibitory influences are removed. Infusions of the GABA_A_ antagonist, picrotoxin, into the cerebellar nuclei on daily training sessions reveals that the short-latency CRs are detectable at frequencies that match those of the normal CRs session-by-session and both reach asymptotic levels within four sessions [32]. Thus, in the current experiment, cortical and nuclear manipulations match the time course for the appearance of normal and short-latency responses in other studies. The extent to which these short-latency responses are stimulus-specific is revealed by generalization gradients. CRs decline as CS parameters move away from the trained values. Because CS auditory frequency generalization gradients of these short-latency responses are similar to those of normal CRs in the intact subject [33], they must be generated from within a system that has sufficient discriminatory capacity and it seems unlikely that a general change in nuclear excitability, of the form that has been found *in vitro* [34] could support this capacity. Synaptic plasticity, similar to that seen *in vitro* [35] seems the obvious candidate and, taken together, these two lines of evidence suggest conditioning-related plasticity of the mossy-fibre collateral inputs to the cerebellar nuclei. This possibility has been tested formally by pharmacological block of these glutamatergic inputs, using a combination of AMPA and NMDA receptor antagonists, in subjects that were displaying short-latency, CS-driven responses following a previous application of a GABA_A_ antagonist [30]. Intriguingly, the short latency responses disappeared but they were replaced, in some cases, by small but appropriately timed CS-driven responses. In reaching a final conclusion about the distribution of plasticity across cortical and nuclear levels in eyeblink/NMR conditioning, it will be important to track down the source of these residual CRs. If the GABA_A_ antagonist action was complete, they are evidence for an extracerebellar contribution to accurately timed conditioned responses.

There is now substantial evidence (see [1] for a review) that another form of cerebellum dependent learning – the modification of VOR gain - depends upon plasticity within the cortical flocculus, as first suggested by Ito [3] *and* upon plasticity in the vestibular brainstem, as first suggested by Miles and Lisberger [5]. It is possible that this two-level plasticity, at cortical and cortical-target neurons, is representative of mechanisms for all or many other forms of cerebellum-dependent learning, such as eyeblink/NMR conditioning [6]. Alternatively, plasticity at cortical and cortical-target neurons in the brainstem (or in the cerebellar nuclei), may be partitioned according to the demands of the task and the motor system plant. In an earlier study of primate VOR calibration Raymond and Lisberger modelled electrophysiological data to deal with learning in the cerebellar cortex [36]. A recent model by Porrill and Dean [7] builds directly upon their conclusions about cortical learning (and so is supported by their actual data), in order to show under what circumstances cortical plasticity *has* to be transferred to the brainstem. It suggests that the 100 ms delay in the retinal slip signal conveyed by climbing fibres to the flocculus would produce learning instabilities at high frequencies, and indeed experimental evidence suggests that these climbing fibres cease responding to retinal slip above 5–10 Hz [37]. However, at such frequencies and above, the complexities of the oculomotor plant caused by elasticity become negligible, so a simple gain change in the vestibular neurons becomes adequate for good VOR performance. The model shows how learning that initially occurs at the cortical level can transfer a gain change to the floccular-target neurons for higher-frequency (>10 Hz) vestibular inputs. Thereby, the system maximizes accuracy by employing the short-latency pathway for high frequency control. One potential disadvantage of this arrangement is that adaptation/learning would be poor or absent at higher frequencies (>10 Hz), though performance would be available throughout the frequency range. Intriguingly, this emergent property of the model provides is a very good fit with the empirical findings and provides a conceptual basis for considering the partitioning of plasticity between cortical and nuclear levels in other forms of learning [7].

If we now consider eyeblink/NMR conditioning, we see that its task demands are different in several respects from those in VOR calibration. In particular, the tasks differ in their response frequencies. As we have shown, a typical single-peak CR with duration of at least 400 ms has 95% of its frequency content below 3.7 Hz, a figure considerably smaller than the highest frequency of the primate VOR, which maintains high precision to 25 Hz. Thus, cortical plasticity would deal with the relatively low-frequencies in eyeblink/NMR conditioned responses, where the complexities produced by elastic properties of the NM response plant demand cortical control. With little requirement for precision in high frequency control, an instructed plasticity at the cerebellar nuclei may be less critical. Thus, eyeblink/NMR conditioning may be especially dependent upon the cortical system, consistent with the current findings, and with a much smaller requirement for nuclear plasticity.

The evidence we present for cortical storage of NMR conditioning memory leaves open the question of its cellular mechanisms but, clearly, they are sensitive to modulation by a GABA_A_ agonist. Long-term depression of parallel fibre (Pf) to Purkinje cell (PC) synapses (Pf-PC LTD) is an obvious candidate [1], [15], [22], [38] but there are multiple potential cortical plasticities [39], [40] including long-term potentiation (LTP) of the Pf synapses to PC and cortical interneuron synapses. Electrophysiology reveals that eyeblink conditioning protocols produce distinct pauses in PC firing rate to presentation of the CS [41] and though such changes could be accounted for via Pf-PC LTD, increased inhibitory drive seems more likely, given that a high proportion of Pf-PC synapses normally may be silent [42], [43]. CS-related, increased inhibitory drive to PCs could be accounted for by LTP of Pf to cortical interneuron synapses and a recent modeling study suggests that bidirectional plasticity at these and at Pf to PC synapses optimizes learning with the noisy signals inherent in Pf input sets [44]. The time course for sensitivity of the consolidation process to the GABA_A_ agonist is from 1–2 hours after each training session [19], a time course strikingly similar to that for monoamine effects in consolidation of other forms of memory [45], [46]. In current work, we find that consolidation of eyeblink/NMR conditioning is sensitive to a cortically-applied noradrenergic antagonist with a time course identical to that seen previously with the GABA_A_ agonist (Kellett and Yeo, unpublished observations). We suggest that a full description of the cellular processes that underlie this form of cerebellar learning await analyses that extend into post-training temporal windows.

## Methods

All procedures were approved by the local ethical review panel of University College London and were in accordance with the UK Home Office Animals (Scientific Procedures) Act under the provision of licence PPL70/6297.

### Surgery

39 male pigmented Murex rabbits (2.2–4.0 kg) were each implanted with one guide cannula directed to cortical lobule HVI and another to the anterior interpositus nucleus (AIP). Subjects were anaesthetised with fentanyl/fluanisone (0.315/10 mg ml^−1^; Hypnorm 0.4 ml kg^−1^ i.m.) supplemented with diazepam (0.4 mg kg^−1^ i.v.), given antibiotic and analgesic cover (enrofloxacin 5 mg kg^−1^ s.c. and meloxicam 0.3 mg kg^−1^ s.c., respectively) and intubated. Subsequently each subject received an infusion of 20% mannitol (10–15 ml kg^−1^ i.v.; 2 ml min^−1^), and was placed in a stereotaxic instrument, with bregma 4.2 mm below lambda. Anaesthesia was maintained with isoflurane (1.5–2.5% in 33% N_2_0, 66% 0_2_). Depth of anaesthesia was monitored by the stability of respiratory rate and the absence of withdrawal reflexes. Under aseptic conditions, the scalp was reflected laterally and a craniotomy performed to expose part of the right cerebellar cortex. The dura was cut and reflected, and cerebellar lobule HVI identified by visual inspection. A 26G stainless steel cannula (Plastics One, Roanoke, VA; length 15 mm below pedestal) was inserted 1.5–2.5 mm below the surface of the medial lobule of HVI, using a compound angle of ∼15° in the coronal plane and ∼10° in the sagittal plane. A second cannula was directed stereotaxically to AIP (AP 4.5 mm, ML 4.0 mm, DV 13.5 mm, relative to lambda). The brain surface was covered in gelatin sponge, and the guide cannulae fixed to the skull using cyanoacrylate and cranioplastic cement. The scalp was sutured around the implant, and 33G dummy cannulae were used to seal the guide cannulae. Postoperatively, subjects received glucose/saline (5%/0.9%; 20 ml kg^−1^ s.c.) and buprenorphine (0.05 mg kg^−1^ i.m.), followed by 3 days of antibiotic (enrofloxacin, 5 mg kg^−1^ s.c.) and analgesic cover (1 day of buprenorphine, 0.05 mg kg^−1^ i.m., followed by 2 days of meloxicam, 0.3 mg kg^−1^ s.c.). All subjects were housed individually, allowed food and water *ad libitum*, and maintained on a 12 hr light/dark cycle for 1 week before and after surgery, and throughout the experiment.

### Conditioning protocols

The apparatus and techniques used for conditioning experiments were similar to those first developed by Gormezano et al. [47] and have been described previously [48]. In each subject a monofilament loop was sutured into the right nictitating membrane under local anaesthesia (proxymetacaine hydrochloride, 0.5% w/v). Subjects were held in a close-fitting Perspex restraining stock, and a low-torque potentiometer (Variohm Model 2200, Towcester, UK) was attached to the head by clips around the ears and muzzle. Each subject was placed in a ventilated, sound-attenuating chamber facing a centrally mounted loudspeaker. The conditioned stimulus (CS) was a 1 kHz sine wave tone of 410 msec duration and an intensity of 81 dB(A-scale). Background noise produced by ventilation fans was 58 dB (A-scale). The unconditioned stimulus (US) was periorbital electrical stimulation (60 msec train of three biphasic pulses of intensity 1.5 mA) delivered through stainless steel clips attached to the skin, one immediately behind the temporal canthus of the eye, the other immediately below the center of the lower eyelid. On paired trials the interstimulus interval (ISI) between the CS and US onset was 350 msec. The inter-trial interval was randomly selected between 25 and 35 sec.

### Experimental Design

The experimental design is summarized in Figure 2.

#### Habituation session

Before conditioning training commenced, a single habituation session of 25 min allowed adaptation to the novel environment of the chamber. During this period each subject was placed in the restraining stock within the conditioning chamber and the nictitating membrane transducer was fitted. The periorbital clips were attached, but the US and CS were not presented.

#### Conditioning sessions

Each conditioning session consisted of 50 trials. In 45 trials the CS and US were paired, and in 5 trials the CS was presented alone. A CS-alone trial was presented on every 10th trial. The acquisition training consisted of four phases with 3 d between each phase. All conditioning sessions were once-per-day so, within each Phase, the intersession interval was 24 hours. Subjects were randomly assigned to either the experimental group (receiving muscimol infusions) or the control group (receiving vehicle infusions).

- *Phase 1*. All subjects received four daily sessions of acquisition training. Immediately after each session, experimental subjects received infusions of muscimol to cortex (7 nmol in 2 µl) and nuclei (7 nmol in 1 µl), and control subjects received infusion of vehicle (154 mM NaCl; 2 µl and 1 µl to cortex and nuclei, respectively). All infusions were delivered simultaneously over 2 min, *via* 33G injectors, with the tip protruding 1.0–1.5 mm beyond the end of the guide cannula. The injector was left *in situ* for 10 min to aid drug diffusion.
- *Phase 2*. All subjects received four daily sessions of training with no post-training infusions.
- *Phase 3*. Experimental subjects that had received muscimol in Phase 1 received four more daily sessions without post-training infusions. Control subjects received four daily sessions of training, each followed immediately by muscimol infusions to cortex and nuclei (7 nmol in 2 µl and 1 µl, respectively) to assess the effects of post-training administration of muscimol on maintenance of established CRs.
- *Phase 4*. Control subjects received an additional four daily sessions of training with no drug, to assess any lasting effects of the post-training infusions in Phase 3.
- *Phase 5 – Performance testing*. Essential for the study was that the relevant eyeblink control regions of both the cerebellar cortex and the cerebellar nuclei were fully inactivated in the critical experimental group. This was established retrospectively in the final Phase 5, in which performance tests established the efficacy of the infusions. By this time all subjects had reached asymptotic levels of CR performance, so reinfusion of muscimol at the cortical location and then, in another session, at the nuclear location revealed the infusion effects at each site. From previous work [49], we know that muscimol infusion in the correct part of HVI or, in different subjects, in the AIP will fully block CR performance.

In previous work, we used autoradiography to track radiolabelled muscimol infusions and establish the maximum extents of drug spread in subjects implanted with a guide cannula either to the cerebellar cortex or to the nuclei [18]. This method revealed whether cortical or nuclear muscimol infusions could block CR performance without drug migration to the other site. Here, however, the problem was different. It was essential to establish that there had been simultaneous, full cortical and nuclear inactivations in each subject. During the critical Phase 1, it was of no consequence whether there had been migration between the sites, only that each was fully inactivated. However, migration between the sites during Phase 5 performance testing would confound a judgment about the extent of inactivation at either site. Compared with earlier, single cannula studies, such migration could be more significant because here there were two cannulae, closely spaced and directed to the two target areas. These placements potentially provided a continuous channel between the sites via the interlobular fissures and increased the probability of migration of the applied drug between the nuclear and cortical sites. To resolve this confound, we used additional testing. We have previously shown that intracortical infusions of the AMPA-kainate receptor antagonist CNQX produce complete block of CR expression [12], [49] but similar infusions directly into critical regions of the cerebellar nuclei have no significant effects upon CR frequency or topography [49]. We took advantage of this dissociation by performance testing all candidate subjects (i.e. those that showed appropriate performance blocks with muscimol) with CNQX infusions in their cortical and, in separate test sessions, in their nuclear infusion sites. Subjects with a rapid (within 5 minutes) and complete block of CRs were deemed to have cortical infusion placements sufficiently close to the cortical eyeblink control regions that their muscimol infusions would have been cortically sufficient – with our without migration to the nuclei. Additionally, we required that CNQX infusions in the nuclei would not produce significant changes in CR expression. Such changes would suggest that nuclear muscimol infusions in these subjects could have been incomplete and exerting their influence by migration to the cortex. Subjects that passed both the muscimol and CNQX performance testing were admitted to the Cortex+Nucleus group.

In order to test whether the muscimol infusions in Phase 1 or 3 had been in appropriate locations and of sufficient concentration fully to inactivate the critical eyeblink control regions during the consolidation time-window, in Phase 5 their efficacy in blocking the performance of established CRs was tested. On Day 1 of Phase 5, the effects of muscimol infusion to cortex only were tested. The test began with a baseline session of 20 trials (18 paired CS–US trials and 2 unpaired CS trials). Muscimol (same concentration, volume and rate as in Phase 1 or 3) was then infused into cortex only. Immediately after the infusion, a 100-trial session was started (90 paired CS-US trials and 10 unpaired CS trials). Similar sessions of 20 trials were then given at 2, 4 and 24 hr after the infusion. In this way, the effects of muscimol infusion were assessed throughout its time-course. On Day 2 of Phase 5, after a 20-trial baseline session, muscimol was infused into nuclei only (same concentration, volume and rate as in Phase 1 or 3), followed by 100 trials immediately, and 20 trials at 2, 4, 6 and 24 hr, respectively. On Day 3, after a 20-trial baseline, the AMPA/kainate receptor antagonist CNQX (6 nmol in 2 µl over 2 min) was infused into cortex only, followed immediately by a 100-trial session. CNQX disrupts performance of CRs when infused into the critical eyeblink zone of lobule HVI, and the dose used in the present experiments has been previously demonstrated as effective in revealing proximity of the cannula tip to the eyeblink control zone. Importantly, CNQX (unlike muscimol) does not affect performance of CRs when infused into AIP. Hence this test confirms that each cortical muscimol infusion acted at its intended target, rather than remotely at the nuclear site. Lastly, on Day 4, CNQX (6 nmol in 1 µl over 2 min) was infused into nuclei (after a 20-trial baseline), followed immediately by a 100-trial session. This test assesses whether muscimol infusions to the nuclei exerted any effects *via* spread to the cortex.

### Histology

At the end of the experiment, infusion sites were marked with 1% pontamine sky blue dye (2 µl to cortex and 1 µl to nuclei). Subjects were given heparin (5000 IU i.v.) and an overdose of pentobarbital sodium (90 mg kg^−1^ i.v.), and transcardially perfused with 0.9% saline (1 litre) followed by 4% formaldehyde in 0.1 M phosphate buffer (2 litres). The brain was removed, cryoprotected in 20% sucrose, and embedded in 10% gelatin. 50 µm serial transverse frozen sections were cut and mounted. One series was stained with cresyl violet, and examined under a light microscope for evidence of cannula damage, gliosis, Purkinje and granule cell loss. Another series was lightly stained with neutral red to reveal the extent of pontamine staining. Infusion sites were reconstructed on standard transverse diagrams.

### Data analysis

A CR was defined as an NMR within the CS–US interval with amplitude ≥0.5 mm and with onset latency >35 msec from CS onset [48]. CR frequency (% CRs) and onset latency was calculated for all trials throughout the conditioning sessions. CR amplitude and latency-to-peak was calculated for unpaired (CS alone) trials only.

### Allocation of Groups by Performance Testing and Histological Examination

A cortical cannula placement was considered on-target only if both muscimol and CNQX infusions produced a suitably rapid and profound abolition of CRs during Phase 5 Performance Testing (demonstrating that muscimol was effective at inactivating cortex within the temporal window vital for consolidation, but without significantly spreading to nuclei). Likewise a nuclear cannula placement was considered on-target only if muscimol succeeded, but CNQX failed, to abolish CRs (demonstrating that muscimol was effective at inactivating nuclei without significantly spreading to cortex).

For admission to the Cortex+Nucleus group, subjects must have satisfied the following criteria: (1) Both cortical and nuclear muscimol abolish CRs for at least one 10-trial block, and to ≤10% within 50 mins of infusion. (2) Cortical CNQX must abolish CRs within 10 min of infusion. (3) Nuclear CNQX must not reduce CRs below 80% during 50 min. (4) The onset of cortical muscimol effects (time to first 10-trial block of 0% CRs) must not be faster than the onset of nuclear muscimol effects (to ensure that the cerebellar nuclei were never in a state of disinhibition during the post-training period) (5) Histological examination must show the centre of the infusions to be within or in the vicinity of HVI and AIP, respectively, with no major cannulation damage.

Subjects in the group Incomplete group met the same criteria as the Cortex+Nucleus group, except that the effects of CNQX infused into cortex were slower in this group, reducing CR frequency to <50% for at least one block.

Subjects in the group ‘Nucleus Only’ had fully effective infusions of muscimol (with ineffective nuclear infusions of CNQX) at the nuclear cannula, but failed to meet the criteria for effective cortical infusion, based upon Performance Testing with either muscimol, or CNQX, or both.

Subjects in the group ‘Off-Target’ failed to meet the criteria for both cortical and nuclear infusions in Performance Testing.

The control (saline) group consisted entirely of subjects who met the criteria for the Cortex+Nucleus or Incomplete groups, but received only saline infusions in Phase 1.

### Drugs & Solutions

Drugs were obtained from the following sources: muscimol hydrobromide from Sigma (Poole, UK), CNQX disodium salt (6-Cyano-7-nitroquinoxaline-2,3-dione disodium) from Tocris (Bristol, UK) or Ascent Scientific (Weston-super-Mare, UK).

### Frequency content of the CR drive signal

Our previous analysis and modelling of NM position and the electromyogram (EMG) of the retractor bulbi muscle that drives the NM response in rabbits, revealed that CR profiles are single-peaked with a typical duration of at least 400 ms with ISIs of 350 ms [21], as used here. The analysis suggests that the CR derives from a neural control signal to the motoneuron pool that has a Gaussian profile, and for an ISI of 350 ms the standard deviation (σ) of the Gaussian is about 130 ms (see [21], Figure 11).

To determine the frequency component content of this neural control signal for the CR, we performed Fourier analysis of this calculated Gaussian:

A temporal Gaussian profile:
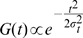
has a Fourier transform which is also a Gaussian:
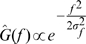
Their widths (standard deviations) are related by:
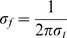
So, for our previously modelled Gaussian CR drive, 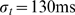 implying that 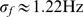, hence 99% of the frequency content is within 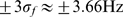.
